# Association of 5p15.2 and 15q14 with high myopia in Tujia and Miao Chinese populations

**DOI:** 10.1186/s12886-020-01516-8

**Published:** 2020-06-26

**Authors:** Junwen Wang, Fang Liu, Xiusheng Song, Tuo Li

**Affiliations:** 1 Department of Hubei Minzu University Affiliated Enshi Clinical Medical School, The Central Hospital of Enshi Tujia And Miao Autonomous Prefecture, No.158, Wuyang Road, Enshi, 445000 Hubei Provence China; 2 Department of Eye Centre, Renmin Hospital of Wuhan University, Wuhan, 430060 Hubei China

**Keywords:** Myopia, Single-nucleotide polymorphism, 5p15.2 region, 15q14 region, Association study

## Abstract

**Background:**

The polymorphisms rs6885224 and rs634990 have been reported to be associated with high myopia in many populations. As there is still no report on whether these two SNPs are associated with myopia in the Tujia and Miao minority areas of China, we conducted a replication study to evaluate the association of single-nucleotide polymorphisms in the regions 5p15.2 and 15q14 with high myopia in Tujia and Miao Chinese populations.

**Methods:**

We performed a comprehensive meta-analysis of 5831 cases and 7055 controls to assess whether rs6885224 in the 5p15.2 region and rs634990 in the 15q14 region are associated with high myopia. Our replication study enrolled 804 individuals. Genomic DNA was extracted from venous leukocytes, and these two SNPs were genotyped by Sanger sequencing. Allele and genotype frequencies were analysed using *χ*^2^ tests, and ORs and 95% CIs were calculated.

**Results:**

According to the results of the meta-analysis, rs6885224 in the *CTNND2* gene showed no association with myopia [*p* = 0.222, OR = 1.154, 95% CI (0.917–1.452)]. Conversely, rs634990 in the 15q14 region did exhibit a significant correlation with myopia [*p* = 7.270 × 10^− 7^, OR = 0.817, 95% CI (0.754–0.885)]. In our replication study, no association with high myopia in the Tujia and Miao populations was found for rs634990 or rs6885224. The following were obtained by allele frequency analysis: rs6885224, *p* = 0.175, OR = 0.845, and 95% CI = 0.662–1.078; rs634990, *p* = 0.087, OR = 0.84, and the 95% CI = 0.687–1.026. Genotype frequency analysis yielded *p* = 0.376 for rs6885224 and *p* = 0.243 for rs634990.

**Conclusions:**

Our meta-analysis results show that rs634990 was significantly associated with myopia but that rs6885224 was not. Nevertheless, in our replication study, these two SNPs showed no association with myopia in the Tujia and Miao Chinese populations. This is the first report involving Tujia and Miao ethnic groups from Enshi minority areas. However, the sample size needs to be expanded and more stringent inclusion and exclusion criteria need to be formulated to verify the findings.

## Background

Myopia is a common and frequently occurring disease, with a high prevalence in both children and adults. Myopia is a type of refractive error that is mainly determined by corneal curvature, lens adjustment and axial length [1]. Myopia is prevalent worldwide, at approximately 28.3% [2]. Furthermore, the situation is severe in East Asia, especially among students and young adults, with a prevalence of almost 90% [2–9]. A recent meta-analysis showed that the number of patients with myopia worldwide has increased from 1406 million in 2000 to 1950 million in 2010; it is predicted that the number of myopia patients will reach 4758 million by 2050 [10]. Myopia can be classified as follows: high myopia, with a spherical equivalent refraction equal to or less than − 6 D; moderate myopia, with a spherical equivalent refraction between − 6 D and − 3 D; and low myopia, with a spherical equivalent refraction equal to or greater than − 3 D. It is worth noting that high myopia is a pathological condition that can lead to many diseases, such as presenile cataracts, glaucoma, macular degeneration, retinal detachment and posterior scleral staphyloma, which are the leading causes of blindness in high myopia [11–15].

Currently, it is believed that the occurrence of myopia is influenced by both environmental and genetic factors [16–19]. Near work, socioeconomic and educational pressures, and reduced time spent outdoors have all been linked to myopia [20–22]. Although the molecular genetic mechanisms involved in the development of myopia are not well understood, many studies, such as genome-wide association studies (GWASs) and pedigree analyses, have revealed many single-nucleotide polymorphisms (SNPs) in different chromosomal regions that are associated with myopia [23–25]. In addition, linkage analysis has mapped approximately 26 Mendelian myopia susceptibility loci (MYP1–26) [26–48]. Despite these results, most of the genes important for myopia have not yet been determined.

In 2011, Yi-Ju Li et al. [49]. performed a meta-analysis using the Singapore Cohort Study of the Risk factors for Myopia (SCORM) and the Singapore Prospective Study Program (SP2) genotyped datasets, with a replication study in a Japanese population, and Boyu Lu et al. [50]. recently carried out a case-control study in a Chinese population. Both studies identified a strong association between rs6885224 in the 5p15.2 region and myopia. Another SNP, rs634990, in the 15q14 region has been demonstrated to have a significant association in Dutch, Japanese, Han Chinese and Guangzhou Chinese populations [25, 51–53]. According to the sixth national census in 2010, the Tujia ethnic group comprises a population of approximately 8 million, accounting for approximately 45% of the total population in Enshi Tujia and Miao Autonomous Prefecture [54]. To date, there is no report on whether these two SNPs are associated with myopia in this minority area. Therefore, we conducted a replication study to examine the association in Enshi Tujia and Miao Autonomous Prefecture.

## Methods

### Ethics statement

All procedures for this study followed the tenets of the Declaration of Helsinki. The ethics committee of The Central Hospital of Enshi Autonomous Prefecture, Enshi, Hubei, China, approved our study. All the patients were informed of the purpose and procedures of the study and provided informed consent in written format prior to the start of the study.

### Meta-analysis

We performed a comprehensive meta-analysis following the Cochrane Handbook to assess whether rs6885224 and/or rs634990 are associated with high myopia. The MEDLINE, EMBASE and Cochrane Library databases were searched for the following keywords: “rs6885224”, “rs634990”, “*CTNND2*”, “*GJD2*”, “*GOLGA8B*”and “myopia”. The search deadline was March 2020. We extracted data, including author, country, year, study design, ethnicity of the subjects, sex, genotyping method and number of alleles and genotypes in cases and controls or the odds ratio (OR) and 95% confidence interval (95% CI), from the included literature. We used these data to perform a comprehensive meta-analysis by using Comprehensive Meta-Analysis Software Version 2.0 (Copyright©2006–2019 Biostat, Inc.). Sensitivity analysis was completed by the “One Study Remove” program of the software. Potential publication bias was assessed using funnel plots and fail-safe N.

### Patients

A total of 804 unrelated subjects recruited from The Central Hospital of Enshi Autonomous Prefecture were enrolled in our study, including 322 healthy controls and 482 high myopia cases. The patients all belonged to the Tujia and Miao ethnic groups. The criteria for the high myopia group were as follows: 1. a spherical equivalent refraction ≤ − 6.0 D; and 2. exclusion of other known ocular or systemic diseases. The criteria for the control group were as follows: 1. a spherical equivalent refraction between − 0.5 D and + 1.0 D and best unaided visual acuity ≥0.8; and 2. exclusion of other known ocular or systemic diseases. The patients were tested for visual acuity and refractive error by autorefraction (Topcon KR-8000, Paramus, NJ, USA) before being enrolled in the study. The case group also underwent ocular biometric axial length examination using IOL Master (Carl Zeiss Meditec AG, Jena, Germany), fundus photography (Canon CF-60UD, Tokyo, Japan) and optical coherence tomography (Heidelberg Engineering HRA + OCT, Heidelberg, Germany).

### DNA extraction

All subjects provided 5 ml of venous blood, which was drawn from the cubital vein. Genomic DNA for all patients and some of the control participants was isolated from leukocytes using the phenol-chloroform method [55]. For the other groups, genomic DNA was extracted from leukocytes using a blood DNA extraction kit (Promega, Madison, Wisconsin, USA) and stored in TE buffer.

### Genotyping

The two SNPs (rs6885224 and rs634990) were genotyped by Sanger sequencing. The Primer3 online tool (http://primer3.ut.ee) was used to design primers for amplification. For rs6885224, the forward primer was 5′-TGGGTGGATGGCTAATGTCA-3′, and the reverse was 5′-TCTTCATCAAGGTTGCTTTGCT-3′; for rs634990, the forward primer was 5′-GCTCAGTGATGCTTGAAGGA-3′, and the reverse was 5′-AGCTTGGAAAACCTTGTGCT-3′. The target fragment was amplified by polymerase chain reaction. The purified amplicons were sequenced with an ABI BigDye Terminator v3.1 Cycle Sequencing kit using an ABI 3100 Genetic Analyzer (Applied Biosystems, Foster City, CA, USA). The sequencing results were compared with consensus sequences (National Center for Biotechnology Information, GRCh37.p13 NC_000005.9 and NC_000015.9) using the SeqMan program of DNAStar software (DNAStar Inc., Madison, WI, USA).

### Statistical analysis

Statistical analyses were performed using a commercial statistical software program (SPSS ver. 25.0; SPSS Science, Chicago, IL, USA). We applied *χ*^2^ tests to evaluate Hardy-Weinberg equilibrium (HWE) for the two SNPs in the case and control groups. The frequencies of alleles and genotypes in the case and control groups were tested by using *χ*^2^ tests; additionally, ORs and 95% CIs were calculated. A two-tailed *p* value of < 0.05 was considered statistically significant.

## Results

**Fig. 1:**
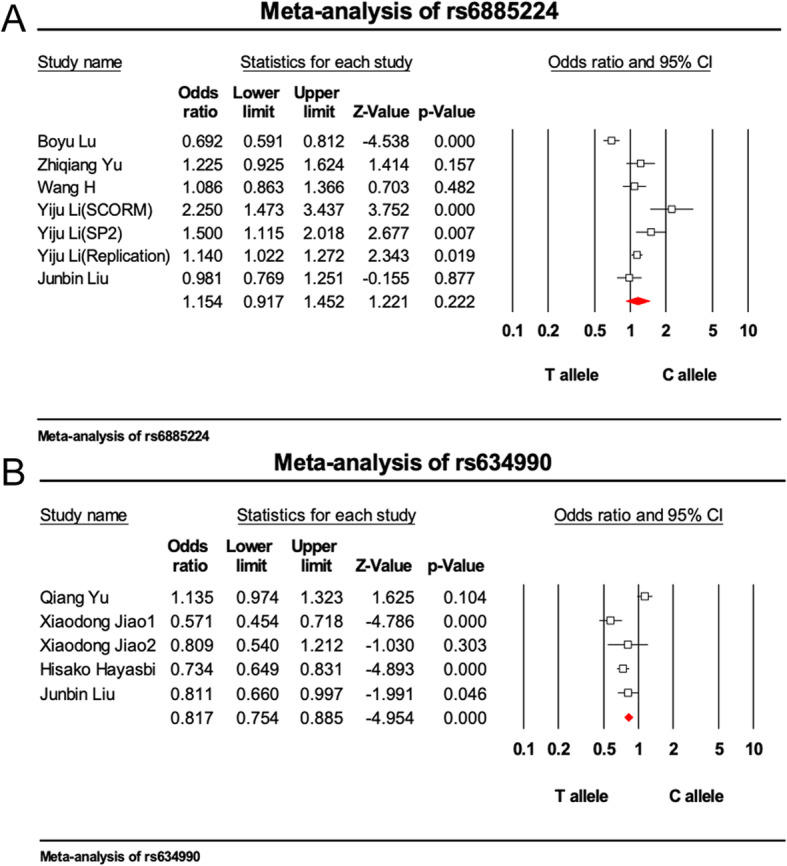
Meta-analysis of association between SNP and myopia. **a**: rs6885224, **b**: rs634990. The OR of each study is shown with a square, the pooled OR is shown with a red diamond

**Fig. 2:**
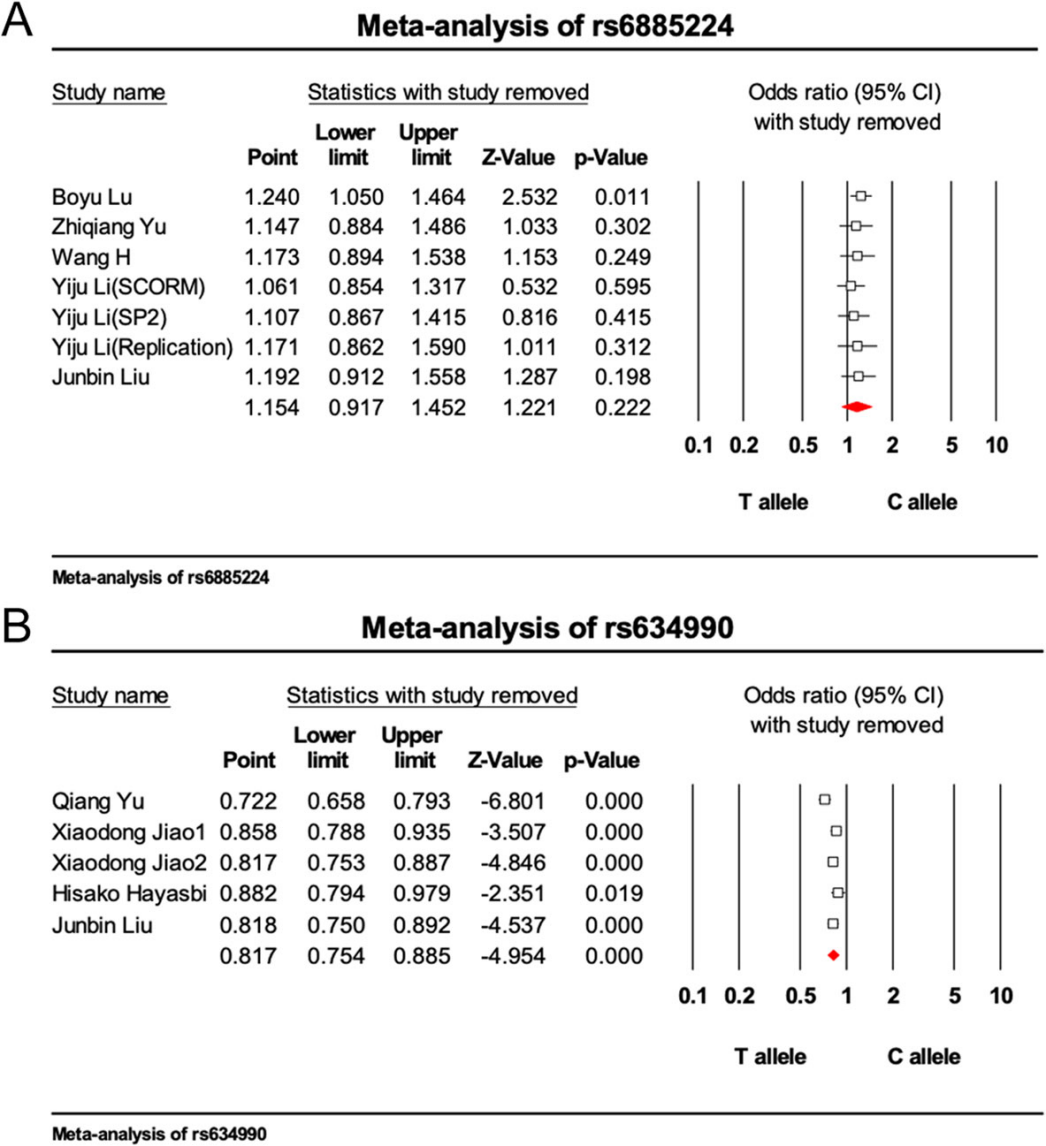
Sensitivity analysis for the polymorphisms. **a**: rs6885225, **b**: rs634990. Each square represents the pooled estimate of the remaining studies after the study is removed, the red diamond represents the pooled estimate of not removing any study

**Fig. 3:**
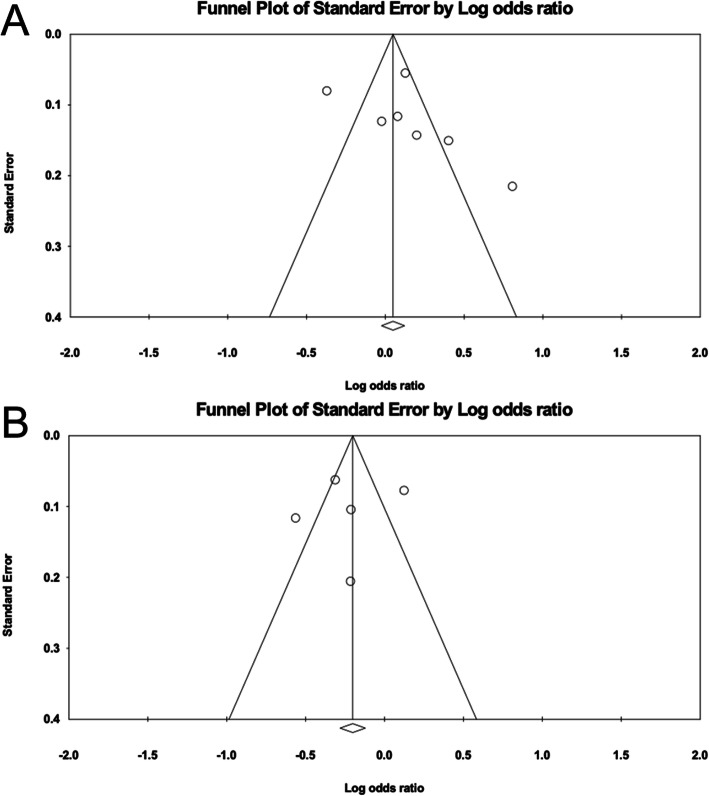
Funnel plots of publication bias analysis for the polymorphisms. **a**: rs6885224, **b**: rs634990. Each small circle represents a study

The main features of the studies included in the meta-analysis are shown in Table 1; 8 published English-language studies from among 11 studies, including 5831 cases and 7055 controls, were evaluated [49–53, 56–58].. According to the results of the meta-analysis, rs6885224 in the *CTNND2* gene showed no association with myopia [*p* = 0.222, OR = 1.154, 95% CI (0.917–1.452)], whereas rs634990 in the 15q14 region did display a significant association with myopia [*p* = 7.270 × 10^− 7^, OR = 0.817, 95% CI (0.754–0.885)] (Fig. 1, Additional file 1). For sensitivity analysis, “One Study Remove” was invoked, deleting each included study step by step, and based on this analysis, no single study significantly changed the pooled estimate when it was removed. (Fig. 2) In two funnel plots, the scatters representing each included study were almost all distributed in the middle and upper part of the inverted funnel. (Fig. 3) The fail-safe N test revealed Z = 2.342 and *p* = 0.019 for rs6885224 and Z = -4.952 and *p* = 7.345 × 10^− 7^ for rs634990. All these results indicate no significant publication bias among the included studies.

**Table 1:** Characteristics of including studies for meta-analysis

Author	Country	Year	Region	Gene	SNP	Age		Case		Control	
						Case	Control	Male	Female	Male	Female
Boyu Lu [50]	China	2011	5p15.2	*CTNND2*	rs6885224	18.53 ± 6.64	24.49 ± 2.82	593	610	558	397
Zhiqiang Yu [56]	China	2012	5p15.2	*CTNND2*	rs6885224	27.50 ± 17.10	41.30 ± 12.40	158	164	168	142
Wang H [57]	China	2016	5p15.2	*CTNND2*	rs6885224	65.72 ± 7.49	59.37 ± 4.06	153	277	165	265
Yiju Li (SCORM) [49]	USA	2011	5p15.2	*CTNND2*	rs6885224	10.83 ± 0.83		65		238	
Yiju Li (SP2) [49]	USA	2011	5p15.2	*CTNND2*	rs6885224	47.90 ± 11.18		222		455	
Yiju Li (Replication) [49]	USA	2011	5p15.2	*CTNND2*	rs6885224	/		959		2128	
Junbin Liu [58]	China	2019	5p15.2/15q14	*CTNND2*	rs6885224/rs634990	29.8 ± 15.8	73.2 ± 8.3	297	291	138	128
Yu Qiang^a^ [53]	China	2014	15q14	/	rs634990	36.00 ± 14.95	42.5 ± 13.30	200	321	139	113
							31.0 ± 10.66			385	303
Xiaodong Jiao 1 [52]	China	2012	15q14	/	rs634990	22.19 ± 1.67	21.66 ± 1.54	148	152	196	112
Xiaodong Jiao 2 [52]	China	2012	15q14	/	rs634990	21.80 ± 1.27	21.68 ± 1.30	63	33	63	33
Hisako Hayasbi [51]	Japan	2011	15q14	/	rs634990	57.57 ± 14.57	38.81 ± 11.83	377	748	573	356

^a^In this study, the control group consisted of two parts

The basic information of the study participants is shown in Table 2. A total of 804 participants were enrolled in the study, including 482 patients with high myopia and 322 normal controls. Of the total, 758 belonged to the Tujia ethnic group, and the remaining 46 belonged to the Miao ethnic group. The axial length in the case group ranged from 26.0 mm to 37.46 mm, with a mean ± standard deviation (SD) of 28.58 ± 2.27 mm.

**Table 2:** Basic information of the participants

Group	Patient, n	Age, mean ± SD	Gander, n (%)		Nation, n (%)		Axial length, mm ± SD	
			Male	Female	Tujia	Miao	OD	OS
Case	482	38.79 ± 18.45	237(49.17)	245(50.83)	453(93.98)	29(6.02)	28.62 ± 2.39	28.54 ± 2.14
Control	322	41.43 ± 11.21	184(57.14)	138(42.86)	305(94.72)	17(5.28)	/	

Allele frequency analysis indicated the following: *p* = 0.175, OR = 0.845, and 95% CI = 0.662–1.078 for rs6885224, and *p* = 0.087, OR = 0.84, and 95% CI = 0.687–1.026 for rs634990 (Table 3). In the genotype frequency analysis, *p* = 0.376 and *p* = 0.243 were obtained for rs6885224 and rs634990, respectively. The two SNPs were in HWE in each group (*p* > 0.05) (Table 4). Therefore, we conclude that although there was a suggestive trend towards an association, neither rs6885224 in 5p15.2 nor rs634990 in 15q14 showed significant differences in allele and genotype frequencies between the high myopia and control groups.

**Table 3:** The allele frequencies of the two SNPs

SNP	Allele	Group	Patient	Allele		*P*	OR	95%CI	Minor Allele	MAF	East Asian	
				C	T						1000G	gnomAD
rs6885224	C > T	Case	482	190	774	0.175	0.845	0.662–1.078	C	0.197	0.239	0.228
		Control	322	145	499					0.225		
rs634990	T > C	Case	482	473	491	0.087	0.84	0.687–1.026	C	0.491	0.465	0.453
		Control	322	288	356					0.447		

*MAF* minor allele frequency; *1000G* 1000 Genome data

**Table 4:** Genotyping and HWE of the two SNPs

SNP	Allele	Group	Patient	Genotype			*P*	HWE	
				CC	CT	TT		Χ^2^	*P*
rs6885224	C > T	Case	482	16	158	308	0.376	0.615	0.433
		Control	322	15	115	192		0.179	0.672
rs634990	T > C	Case	482	120	233	129	0.243	0.520	0.471
		Control	322	67	154	101		0.344	0.557

*HWE* Hardy-Weinberg equilibrium

## Discussion

We performed this comprehensive meta-analysis to assess whether rs6885224 and rs634990 are associated with myopia. Our results showed that rs634990 was significantly associated with myopia but that rs6885224 was not. Nevertheless, we do not recommend a conclusion on this point. Indeed, the development of myopia is affected by both genetic and environmental factors. In general, different results will be obtained for people from different regions and ethnic groups due to genetic heterogeneity. This makes it necessary to conduct verification studies among different ethnicities. However, as there are, to our knowledge, no relevant reports on Tujia or Miao populations, we performed a replication study to assess the correlation of two potential SNPs associated with high myopia in the Tujia and Miao populations. These SNPs have been reported to be associated with high myopia in Han Chinese, Singaporean Chinese, Japanese and Dutch populations [49–53, 56, 57]. The Enshi Tujia and Miao Autonomous Prefecture is the only autonomous prefecture with ethnic minorities in Hubei Province. It is an ethnic minority area mainly characterized by Tujia, Miao, Dong, Bai, Mongolian, Hui and other ethnic minorities, with Tujia and Miao accounting for the majority. The Enshi minority region is located in southwest Hubei Province and is considered a low-income area. Although the quality of life in this area has been somewhat improved by the development of railways and highways in recent years, living habits are still relatively conservative, and migration into and out of the region is limited. No studies to date have been conducted in the populations in this area. According to our results, neither rs6885224 nor rs634990 is associated with high myopia in these population.

**Fig. 4:**
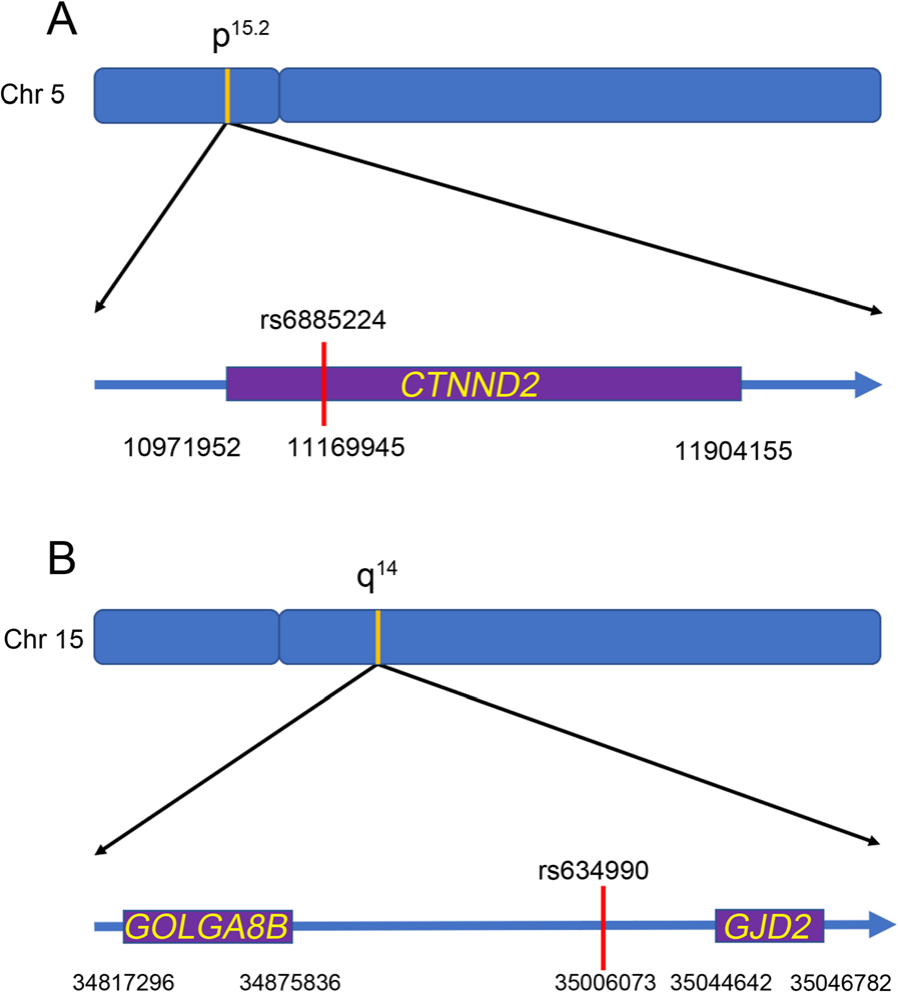
Schematic diagram of SNP location **a**: rs6885224 located in the chromosome 5p15.2 region within the *CTNND2* gene. **b**: rs634990 located in the chromosome 15q14 region, which is the intergenic region near the *GOLGA8B* gene and *GJD2* gene

The SNP rs6885224 is located in region 5p15.2 within the Catenin Delta 2 gene (*CTNND2*), which belongs to the beta-catenin family, is 932 kb in length, and includes 26 exons (Fig. 4-a). This gene encodes an adhesive junction-associated protein in the armadillo/beta-catenin subfamily that is involved in the development of the brain and eyes as well as the development of cancer [59–62]. Expression of the *CTNND2*-encoded protein is stimulated by hepatocyte growth factor, which then promotes the destruction of E-cadherin-based adherens junctions [63]. It was previously reported that this gene is located in a region on the short arm of chromosome 5, and hemizygosity of *CTNND2* is associated with cri du chat syndrome [64]. Furthermore, Matter et al. [65] reported attenuated cortical responses to visual stimulation in 10-week-old mice with homozygous loss of *CTNND2*. Some have speculated that rs6885224 in *CTNND2* might regulate mRNA transcription and affect expression of the gene, thereby affecting the occurrence of myopia [50]. Others have speculated that *CTNND2* may regulate the structure and function of the sclera by breaking down E-cadherins in scleral fibroblasts, which may lead to myopia [56].

The SNP rs634990 in region 15q14 has no corresponding nearby gene. It is located in the intergenic region near the gap junction protein delta-2 gene (*GJD2*) and the golgin A8 family member B gene (*GOLGA8B*) (Fig. 4-b). *GJD2*, also called connexin-36 (CX36) and formerly called GJA9, is located approximately 39 kb downstream of rs634990 and encodes a member of the connexin protein family. Studies have shown that CX36 is present in a number of retinal neurons, including rod photoreceptors, cone bipolar cells and all amacrine cells [66]. The gap junction containing CX36 plays an important role in normal synaptic transmission in the rod pathway [67]. In addition, expression of CX36 contributes to the survival of retinal cells and resistance to injury, and it has been found to play an important role in the electrophysiology of the chicken retina [68, 69]. *GOLGA8B* is a protein-coding gene that may be involved in maintaining Golgi structures. Regardless, the specific function of this gene in the eyes has not been reported, though a study by Solouki et al. [25] examined *GOLGA8B* gene expression in the retinas of postmortem humans and found that *GJD2* was highly expressed nut that *GOLGA8B* was lowly expressed. rs634990 is located in the intergenic region of these two genes and might be related to the expression or function of either gene.

In our study, neither rs634990 nor rs6885224 showed an association with high myopia in the Tujia and Miao populations. Additionally, HWE *p* values > 0.05 were observed for both the experimental and control groups, indicating that the alleles carried by the subjects were in HWE and that the subjects are from a population with random mating and little influx of new genetic material. Although it appears that our sample was reliable, our results differed from those of previous studies [25, 49–52]. The reasons for the conflicting results may be because the pathogenesis of myopia is complex and is influenced by both environmental and genetic factors. First of all, myopia is a multifactorial genetic disease, which is determined by a combination of environment and genetic factors, and neither can be ignored. The genetic backgrounds of the Tujia and Miao populations differ from other ethnic groups in China and the world, and our research is designed to explore these differences in relation to myopia. In fact, few genetic studies have been carried out on the Tujia and Miao populations, and we feel that those differences we have found are worth being further analyzed, even if we cannot completely account for environmental factors due to practical limitations of the study. Secondly, in terms of environmental factors, these environmental factors, such as educational level, near-work, outdoor activities, work in artificial light and the use of digital electronic products, are also important risk factors for myopia. Especially for education level and outdoor activity time, longer education means more near-work and less outdoor activities. Many scholars around the world are doing relevant studies. A large number of studies have shown that overweight learning burden, long hours of near-work and less outdoor activities can increase the risk of disease [70–73]. The Enshi ethnic minority area belongs to poor mountainous area with backward traffic and communication conditions, which prevents us from making return visits to the participants. Nevertheless, we are still trying to get in touch with them. In our efforts, only 52 cases and 26 controls were contacted. The education years of 52 cases are 8.63 ± 3.29 and that of the controls are 6.65 ± 3.71, which means that there is significant difference between the two groups (t = 2.402, *p* = 0.019). Unfortunately, this number of responses is not enough to serve as covariates in our study. Thirdly, there are also some studies that have reported the interaction of genetic and environmental factors on the risk of myopia [71, 72, 74–76]. For example, the study of Fan et al. found that three genome-wide associated loci, *AREG*, *GABRR1* and *PDE10A*, showed strong interaction with education in Asian populations, but this interaction was not significant in European populations [77]. This study not only showed the interaction between genetics and environment, but also showed that the interaction is different in different ethnic groups. Although the SNPs in these studies are different from those we studied, they do suggest that interactions between genetics and environment have impact on the risk of myopia. The study by Pozarickij et al. does show an interaction for the 15q14 region, although not for the specific SNPs we used [74]. For now, no paper has reported on the interaction of genetic and environmental factors in Tujia and Miao populations, so it is worth collecting more data about Tujia and Miao populations for further analysis and research, but that is beyond the scope of this report.

Of course, we have to admit that there are some limitations in our study. First of all, this is a population-based study which requires a large enough sample size to provide more forceful evidence. However, our research involves a relatively small sample size, which leads to the fact that our study evidence seems unconvincing. Secondly, we chose the two SNPs that have been studied on the previous reports. More pathogenic SNPs should be found through GWAS study in the future. What’s more, in future studies, we should include environmental factors such as education years, outdoor activity time and electronic product frequency etc. and conduct stratified analysis on them to give a further analysis on the association between SNP and myopia in Tujia and Miao populations.

## Conclusions

In our replication study, we found that neither rs6885224 in the *CTNND2* gene nor rs634990 in the 15q14 region was associated with high myopia in the Tujia and Miao populations in Enshi Tujia and Miao Autonomous Prefecture. This is the first report involving Tujia and Miao ethnic groups in the Enshi minority areas and provides reference data for future studies needed to verify the study result.

## Supplementary information

**Additional file 1: Supplementary table 1.** Statistics for each study in the meta-analysis of rs6885224, **Supplementary table 2.** Statistics for each study in the meta-analysis of rs634990.

## Data Availability

The datasets used and/or analyzed during the current study are available from the corresponding author on reasonable request.

## Abbreviations

GWASs: Genome-wide association studies
SNPs: Single-nucleotide polymorphisms
SCORM: Singapore Cohort Study of the Risk factors for Myopia
SP2: The Singapore Prospective Study Program
OR: Odds ratio
CI: Confidence interval
HWE: Hardy-Weinberg equilibrium
CTNND2: Catenin Delta 2
GJD2: Gap junction protein delta-2
GOLGA8B: Golgin A8 family member B
CX36: Connexin-36
MAF: Minor allele frequency
