# Supplementary material for: Association of 5p15.2 and 15q14 with high myopia in Tujia and Miao Chinese populations
Source: BMC Ophthalmol. 2020 Jun 26;20:255. doi: 10.1186/s12886-020-01516-8 (PMC7318420; doi:10.1186/s12886-020-01516-8)
Supplement: Supplementary file 1 — Additional file 1: Supplementary table 1. Statistics for each study in the meta-analysis of rs6885224, Supplementary table 2. Statistics for each study in the meta-analysis of rs634990. [file 12886_2020_1516_MOESM1_ESM.zip › Additional file 1.pdf]

Additional file 1

Supplementary table 1: Statistics for each study in the meta-analysis of rs6885224

Study name	Odds ratio	Lower limit	Upper limit	Z-value	P-value
Boyu Lu	0.692	0.591	0.812	-4.538	5.674×10^{-6}
Zhiqiang Yu	1.225	0.925	1.624	1.414	0.157
Wang H	1.086	0.863	1.366	0.703	0.482
Yiju Li (SCORM)	2.250	1.473	3.437	3.752	1.757×10^{-4}
Yiju Li (SP2)	1.500	1.115	2.018	2.677	7.434×10^{-3}
Yiju Li (Replication)	1.140	1.022	1.272	2.343	0.019
Junbin Liu	0.981	0.769	1.251	-0.155	0.877
Pooled estimate	1.154	0.917	1.452	1.221	0.222

Supplementary table 2: Statistics for each study in the meta-analysis of rs634990

Study name	Odds ratio	Lower limit	Upper limit	Z-value	P-value
Qiang Yu	1.135	0.974	1.323	1.625	0.104
Xiaodong Jiao1	0.571	0.454	0.718	-4.786	1.700×10^{-6}
Xiaodong Jiao2	0.809	0.540	1.212	-1.030	0.303
Hisako Hayasbi	0.734	0.649	0.831	-4.893	9.942×10^{-7}
Junbin Liu	0.811	0.660	0.997	-1.991	0.046
Pooled estimate	0.817	0.754	0.885	-4.954	7.270×10^{-7}
